# Reprogramming oral epithelial keratinocytes into a pluripotent phenotype for tissue regeneration

**DOI:** 10.1002/cre2.455

**Published:** 2021-05-22

**Authors:** Fayrouz Bazina, Sabine M. Brouxhon, Stephanos Kyrkanides

**Affiliations:** ^1^ Ph.D. Program in Oral Biology and Pathology, School of Dental Medicine Stony Brook University Stony Brook New York USA; ^2^ Center for Oral Health Research, College of Dentistry University of Kentucky Lexington Kentucky USA; ^3^ Department of Physiology, School of Medicine Stony Brook University Stony Brook New York USA; ^4^ Department of Oral Health Science, College of Dentistry University of Kentucky Lexington Kentucky USA

**Keywords:** dedifferentiation, oral epithelium, stemness, TGFβ

## Abstract

**Objectives:**

We set out to reprogram adult somatic oral epithelial keratinocytes into pluripotent cells for regenerative dentistry.

**Setting and Sample population:**

Immortalized murine oral keratinocyte cell (IMOK) line raised from adult mouse mucosa were cultured in vitro in our studies.

**Materials and Methods:**

Adult murine oral epithelial keratinocytes were chronically treated with TGF‐β1 in vitro, and the expression of Oct4, Nanog, Sox2 and Nestin, as well as specific homeobox Gata and Pax gene family members were investigated.

**Results:**

We documented the induction of stem factors linked with pluripotency and/or the maintenance and regulation of stem‐cell self‐renewal in oral epithelial keratinocytes by TGFβ1. Moreover, we discovered that this TGF‐β1‐induced increase in Oct4, Nanog, Sox2 and Nestin was inhibited by SB431542, suggesting that TGF‐β1 signals via the TGF‐βRI receptor to induce pluripotency and stemness.

**Conclusions:**

Adult oral epithelial keratinocytes treated chronically with TGF‐β1 acquired phenotypic characteristics consistent with pluripotent stem cells, highlighting the facileness of reprogramming adult oral keratinocytes into an unlimited supply of pluripotent stem cells.

## 1 INTRODUCTION

Regenerative dentistry efforts aiming to restore oral, dental and craniofacial tissues impacted by injury or disease is a promising field with future clinical applications. Considerable progress has been made in producing whole teeth, or parts of a tooth, or even biomimetic dental materials (Galler & D'Souza, 2011; Mitsiadis et al., 2015). Although recent advances in the field of regenerative dentistry are promising, one significant limitation continues to be the inadequate supply of cells needed in tissue and organ regeneration (Niibe et al., 2017). Therefore, the purpose of our study is to interrogate the reprogramming potential of adult oral epithelial keratinocytes, which abundantly line the oral cavity, as a source of cells for regenerative dentistry.

Recent studies suggest that adult somatic cells exhibit remarkable plasticity, and can be reprogrammed to acquire pluripotency or stem cell‐like properties (Chakravarti et al., 2014). Such pluripotent cells can then be differentiated towards lineage‐specific cell types so as to replace damaged or diseased tissues and organs (Chakravarti et al., 2014). Pluripotent cells are a transient population of cells that depend on transcription factors, such as Oct4, Nanog and Sox2, to maintain pluripotency, whilst simultaneously repressing lineage specification (Chambers et al., 2007). Octamer‐binding protein 4 (Oct4) was the first master gene shown to be required for pluripotency of murine and human pluripotent stem cells (Zeineddine et al., 2014). As such, Oct4 is well accepted as a core pluripotency factor in association with its partner transcription factors, Nanog and Sox2 (Shi & Jin, 2010). Genetic studies report that the core pluripotent genes, Oct4, Nanog and Sox2, are highly expressed in stem cells (Huang & Garcia‐Godoy, 2017). Moreover, cells losing Oct4 during murine embryonic development were found to differentiate into somatic cells (Pesce & Scholer, 2001). Sox2 further plays an essential role in maintaining cells in this undifferentiated state and facilitating lineage commitment during embryonic development (Carrasco‐Garcia et al., 2016). The unique ability of Sox2 to cooperate with Oct4 at specific binding sites is also critical for reprogramming fully differentiated somatic cells into induced pluripotent cells (Aksoy et al., 2013). Nanog also has been considered another key factor in the generation of iPSCs, as addition of Nanog to Yamanaka factors (Hanna et al., 2009), which include Oct4, Sox2, Klf4, and c‐Myc, has been shown to enhance the reprograming kinetics (Hanna et al., 2009). Yu et al. (2007) demonstrated that Nanog and Oct4, along with Lin28 and Sox2, induced the de‐differentiation of human fibroblasts into pluripotent stem cells, with the full capacity to differentiate into any cell type. In contrast, Nestin belongs to class VI intermediate filament proteins, and is widely used as a marker of stem cells that are capable of multilineage differentiation (Chen et al., 2006).

Transforming growth factor beta (TGF‐β1) is considered a key component of the stem cell niche in many different tissues and appears to play an essential role in modulating epithelial cell plasticity and pluripotency. TGF‐β1 has been reported to upregulate pluripotency transcription factors in adult somatic cell lines in vitro. To this end, Au et al. (2015) showed that TGF‐β1 induced the expression of Oct4 in primary human endometriotic stromal cells. Furthermore, TGF‐β1 upregulated Nestin in human kidney proximal tubules cells (Wen et al., 2012) and in ventricular fibroblasts (Hertig et al., 2017). However, it remains unclear whether TGF‐β1 can induce pluripotency in adult oral epithelial keratinocytes.

In the present study, we investigated whether chronic TGF‐β1 is capable of reprogramming an immortalized murine oral keratinocyte cell (IMOK) line developed from the oral mucosal specimens of adult mice into a phenotype that expresses one or more of the key pluripotency transcription factors, in vitro.

## 2 MATERIALS AND METHODS

### 2.1 Cell culture and reagents

The Immortalized Murine Oral Keratinocytes (IMOK) cell line was kindly donated by Dr. Garrett‐Sinha at SUNY‐Buffalo (Parikh et al., 2008) as part of NIH's Resource Sharing plan for funded investigators. Specifically, the IMOK cells were raised from tissue containing oral epithelium from the buccal and palatal regions were harvested from 6 month old female 129Sv mice. Cells were seeded in serum‐free keratinocyte media (CnT‐02; CELLnTEC; Advanced Cell System) and maintained at 37°C in a humidified chamber containing 5% CO_2_. Upon reaching 40% confluency, cells were treated with 5 ng/ml recombinant mouse TGF‐β1 (Bazina et al., 2021; Valcourt et al., 2005) (R&D System), or equal volumes of vehicle, for 2‐, 4, and 6‐days. For TGF‐βRI inhibitory experiments, the solid anhydrous TGF‐βRI kinase inhibitor SB‐431542 (TOCRIS) was added to the culture media (10 μM final concentration).

### 2.2 Immunofluorescence (IF)

Cells were cultured on Lab‐Tek chamber slides (Nalgene Nunc International) until 40% confluent before treatment with either TGF‐β1 or vehicle control, at the indicated time points. Cells were fixed with 4% formaldehyde and processed for immunofluorescence microscopy (Kyrkanides et al., 2012). Primary antibodies included Oct4 and Nanog (Abcam), Nestin (Novus Biologicals), counter stained with DAPI (Vector Laboratories). Images of cover slipped slides were captured under 594 nm (red) and 350 nm (blue) wavelengths using a BX51 Olympus fluorescent microscope.

### 2.3 Flow cytometry

Samples were prepared as described previously (Al‐Attar et al., 2018). Briefly, harvested cells were fixed with 2% paraformaldehyde, washed with permeabilization buffer (BioLegend) and incubated with primary antibodies: Oct4 (Chemicon), Nanog (Cell Signaling), Sox2 (Santa Cruz Biotechnology), Nestin (Novus Biologicals), and Rabbit IgG (Abcam) and chicken IgY isotype (R&D Systems) controls. After rinsing, cells were incubated with an Alexa flour‐488 goat anti‐rabbit secondary antibody (Molecular Probes), or a FITC goat anti‐chicken IgY (Novus Biologicals) isotype control. Stained samples were read on an LSR‐II cytometer using FACSDiva version 6 (BD Biosciences) and analyzed by FlowJo Software (TreeStar; version 9.5). Data are represented as geometric mean‐fluorescence intensity (GMFI).

### 2.4 Western blotting

Cells were processed for western blotting, as described previously (Brouxhon et al., 2007). Briefly, samples were electrophoretically separated using SDS‐PAGE, and transferred to nitrocellulose membranes. Membranes were blocked with 5% (w/v) nonfat dry milk to block nonspecific binding, and then incubated with TGF‐βRI, TGF‐βRII, Sox2 and β‐actin (Santa Cruz Biotechnology), Oct4 (Chemicon), Nestin (Novus Biologicals), Nanog, TGF‐βRIII, phospho‐specific Smad2, Smad3, Smad2, Smad3, and Smad4 (Cell signaling). Proteins were detected using appropriate HRP‐conjugated secondary antibodies (Santa Cruz Biotechnology). Immunoreactive bands were detected using the Clarity MaxTM Western ECL Substrate (Bio‐Rad), and visualized using the ChemiDoc MP Imaging System (Bio‐Rad). Western blot signals were normalized to β‐actin, using NIH Scion Image. Results are presented as fold increase relative to controls in triplicate experiments.

### 2.5 Cytokine array

The secretion of cytokines was evaluated using a Cytokine Antibody Array C1 Series (RayBiotech), according to the manufacturer's instructions. Briefly, conditioned medium was obtained after culturing cells for 48 h in serum‐free medium at 37°C and 5% CO_2_. Each array was incubated with 1 ml of undiluted medium at 4°C overnight, washed, and then incubated with a biotinylated antibody cocktail, followed by an HRP‐streptavidin incubation. To determine the level of cytokines in the media, spot signal densities were measured using Image Studio™ Lite (LI‐COR Biosciences), and data analysis was performed using the RayBiotech Microsoft® Excel‐based analysis software.

### 2.6 TGF‐β1 ELISA

A commercial ELISA kit for secreted TGF‐β1 (Biolegend) was used to quantify concentrations in cell culture supernatants according to the manufacturer's instructions.

### 2.7 Stem cell gene array

A custom‐plate Prime‐PCR mouse‐stem cell gene array (SAB target list‐ M96; Bio‐Rad) was used to analyze the expression of the stem cell signaling‐associated genes in IMOK cells, treated in the presence or absence of TGF‐β1. RNA was extracted from cells using the RNeasy Mini Kit (Qiagen), and RNA reversed transcribed using the iScript Advanced cDNA Synthesis kit (Bio‐Rad). The reaction mix was prepared using the Advanced Universal SYBR® Green Supermix (Bio‐Rad), per the manufacturer's instructions. For real‐time polymerase chain reaction (qRT‐PCR), a CFX96 Touch Detection System (Bio‐Rad) was used. For data normalization, stable reference genes, such as glyceraldehyde‐3‐phosphate dehydrogenase (GAPDH), was embedded in the plate setup and employed for Δ‐Cq calculations. cDNA templates were assayed in triplicates, and results presented as relative gene expression (ΔΔ‐Cq Expression) in the TGF‐β1 treated groups normalized to the corresponding vehicle control samples.

### 2.8 Statistical analysis

All data are shown as the means ± SEM. To determine statistical differences between or among means, a student's *t*‐test was performed where applicable. For all analyses, a *p*‐value equal to or less than 0.05 was considered statistically significant.

## 3 RESULTS

### 3.1 Characterization of secreted cytokines from naive IMOK cultures in vitro

**FIGURE 1:**
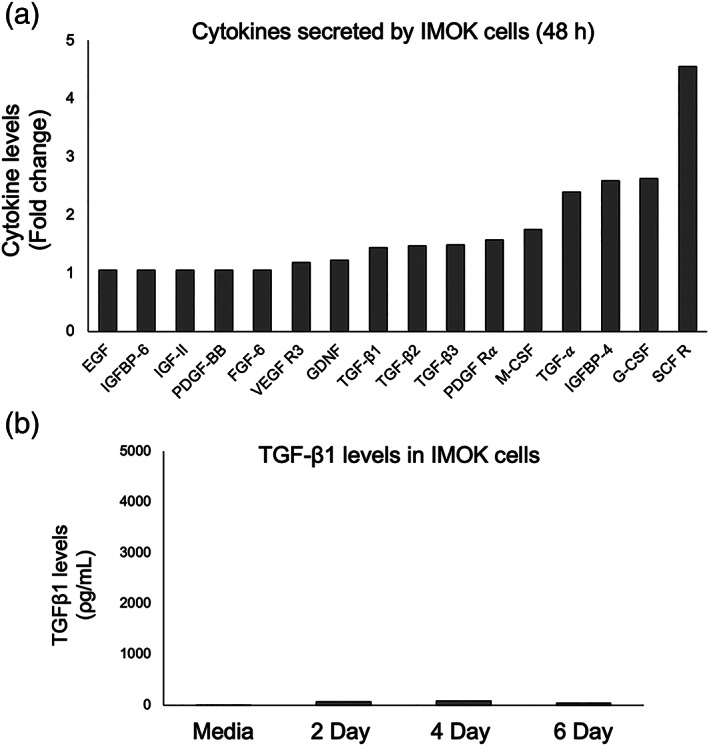
Characterization of cytokines released by naïve IMOK cells. (a) Array analysis of growth factors released in the supernatants of naïve IMOK cells in culture compared to fresh media (control) at 2‐days post seeding (*n =* 2). (b) The concentration of active TGF‐β1 was assessed by ELISA collected from naïve IMOK supernatants at 2‐, 4‐ and 6‐days post seeding. The results are shown as the mean ± SEM in triplicate experiments

We performed a comprehensive analysis of the secretome of IMOK cells at baseline conditions using an antibody‐based ELISA array. Our results show that IMOK cells secrete into the conditioned media, compared to control media, high levels of TGF‐α (2.3‐fold), IGFBP‐4 (Insulin‐like Growth Factor‐Binding Protein 4; 2.5‐fold), G‐CSF (Granulocyte Colony‐Stimulating Factor; 2.6‐fold), and SCFR (Stem Cell Factor Receptor/c‐Kit; 4.5‐fold) (Figure 1a). We further assessed TGF‐β1 levels in conditioned media at the 2‐, 4‐, and 6‐ day time points by ELISA. In agreement with the cytokine array, levels of TGF‐β1 were negligible (~40 pg/ml) at all‐time points examined (Figure 1b), suggesting that IMOK cells constitutively secrete low levels of TGF‐β1 under basal conditions.

### 3.2 Chronic TGF‐β1 induced the upregulation of pluripotency markers in IMOK cells

**FIGURE 2:**
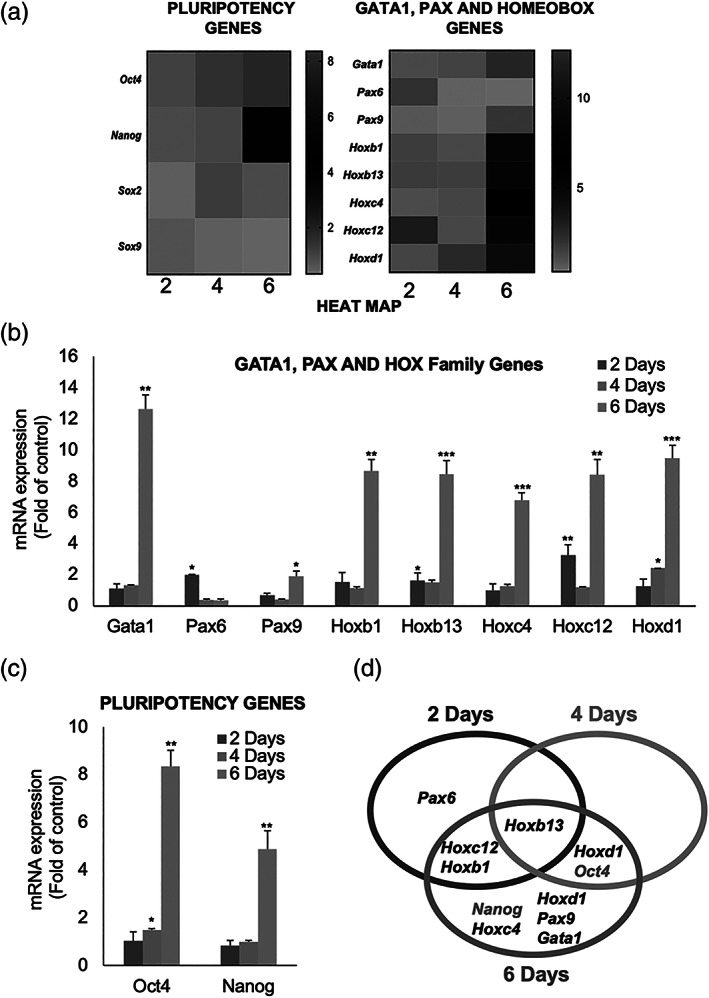
Pluripotency, Gata1 and Homeobox gene changes in IMOK cells after chronic TGF‐β1 treatment. A primePCR stem cells gene array (BioRad) was used to evaluate relative gene expression profiles of IMOK cells after chronic TGF‐β1 treatment. (a) Clustergram demonstrating differentially expressed pluripotency, Gata1 and Homeobox genes in IMOK cells treated with TGF‐β1, or vehicle control, for 2‐, 4‐, and 6‐days after normalizing to GAPDH. Red indicates greater expression, green indicates lower expression Cq > 30. The data of the representative genes were derived from three independent experiments. (b) Bar graph showing relative expression of selected Gata1 and Homeobox genes that have >1.5‐fold increase in the ΔΔCq expression after 2‐, 4‐, and 6‐days chronic treatment with TGF‐β1, versus their corresponding vehicle controls. (c) Gene levels of Oct4 and Nanog at the selected time points. (d) Venn diagram illustrating distinct and overlapping gene expression for the up‐regulated genes in 2‐day (blue), 4‐day (Orange), and 6‐day (Gray) TGF‐β1 treated IMOK cells relative to their vehicle control group. Experiments were conducted in triplicates. Data are presented as mean + SEM; *p < 0.05; **p < 0.01; ***p < 0.001

We characterized the stem cell transcriptome of IMOK cells in response to TGF‐β1 by a quantitative real‐time PrimePCR array (Bio‐Rad; custom‐designed plates) including a panel of stem cell‐associated genes in total RNA extracted from TGF‐β1 treated and vehicle control groups, at the 2‐, 4‐, and 6‐day time points. In our analysis, we selected genes that showed at least a 1.5‐fold up‐regulation by TGF‐β1, compared to vehicle controls. Our results indicate that TGF‐β1 upregulated 10 out of the 29 genes at the time‐points examined. Specifically, TGF‐β1 upregulated Pax6, Hoxb1, Hoxb13, and Hoxc12 genes in 2‐day cultures, while Hoxb13, Hoxd1, and Oct4 (Pou5f1) genes were increased by day 4 (Figure 2a,b). Furthermore, the core pluripotency markers Oct4 (Pou5f1) and Nanog, as well as other stemness‐related genes, including Hoxb1, Hoxb13, Hoxc4, Hoxc12, Hoxd1, Pax9, and Gata1 were significantly increased by day 6 (Figure 2a–c). Notably, only Hoxb13 gene revealed a persistent TGF‐β1‐induced up‐regulation over the three‐time points examined (Figure 2d).

**FIGURE 3:**
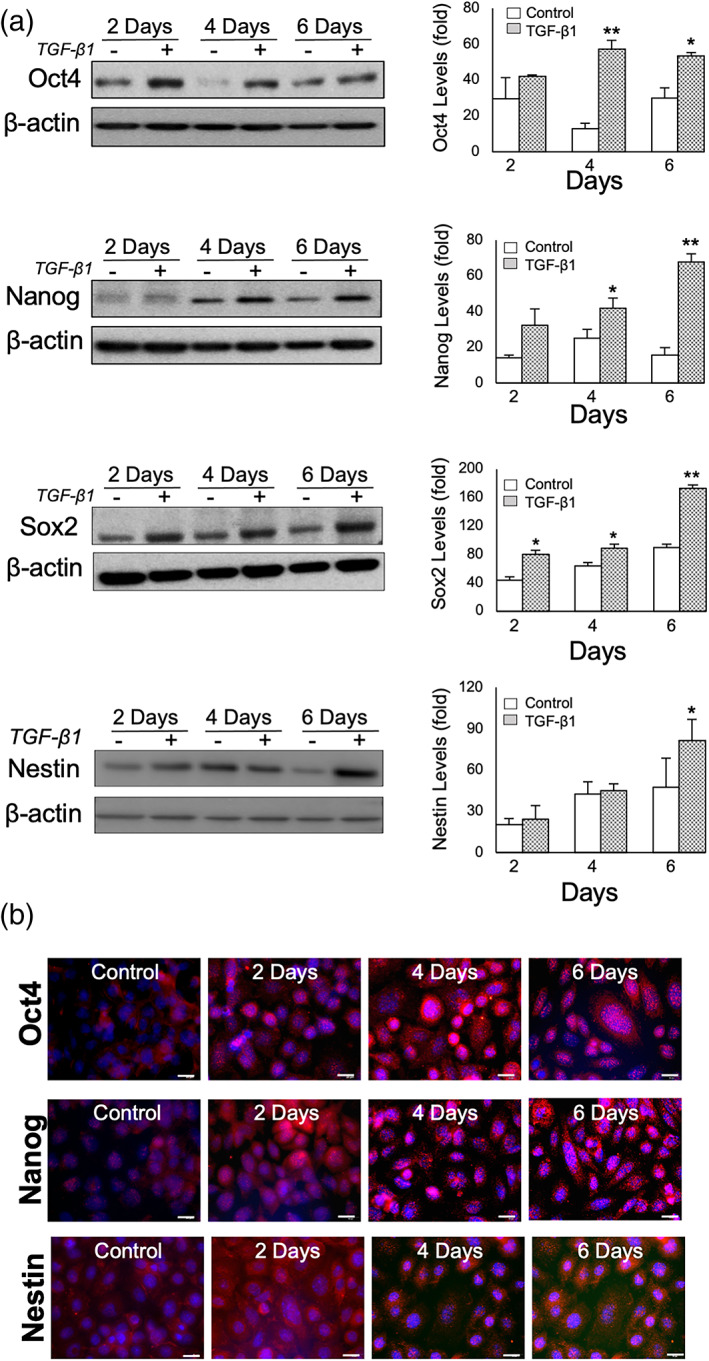
Oct4, Sox2, Nanog and Nestin induction in IMOK cells after chronic TGF‐β1 in vitro. (a) Representative immunoblot and analysis of the expression levels of Oct4, Sox2, Nanog and Nestin in total cell lysates from IMOK cells treated chronically with TGF‐β1 versus vehicle controls at the indicated time points by western immunoblotting. Expression levels were normalized to β‐actin and quantitated by NIH Scion Image. (b) Representative immunofluorescence images for Oct4, Nanog and Nestin (red) in control and TGFβ1‐treated cells at the indicated times. Nuclei were labeled with DAPI (blue). Data are presented as mean + SEM. *p < 0.05, **p < 0.01, versus corresponding control (*n =* 3). Bar = 20 μm

Changes in the stem cell transcription factors Oct4, Nanog, Sox2 and Nestin were next evaluated at the protein level by western blotting, in response to chronic TGF‐β1 treatment in IMOK cells at 2‐, 4‐, and 6‐days in vitro. Densitometric analysis of the immunoreactive bands, normalized to their corresponding loading controls, revealed that Oct4, Nanog, Sox2 and Nestin were significantly increased in response to TGF‐β1 (Figure 3a **)**. Specifically, immunoblot analysis showed a statistically significant increase in Oct4 levels, that peaked at 4‐days. In contrast, Nanog, Sox2 and Nestin peaked at the 6‐day time point. Next, we assessed the spatiotemporal localization of Oct4, Nanog and Nestin after chronic TGF‐β1 treatment by immunofluorescence. Consistent with our western blotting results, we observed an intensification of immunostaining in response to TGF‐β1 treatment over time (Figure 3b). Additionally, Oct4 and Nanog demonstrated strong nuclear staining in the TGF‐β1‐treated cells which peaked at 6‐days, compared to controls. Similarly, Nestin immunostaining was weak in control conditions, but demonstrated a strong TGF‐β1‐induced nuclear and perinuclear localization at 4 days (Figure 3b). Taken together, these data suggest that chronic TGF‐β1 induces phenotypical changes in adult oral keratinocytes associated with the upregulation and nuclear localization of the key master transcription factors Oct4, Nanog, Sox2, as well upregulation of other markers involved in the maintenance of this undifferentiated state and regulation of stem cell self‐renewal.

### 3.3 Expression of TGF‐β1 cognate receptors are induced by TGF‐β1 in IMOK cells

**FIGURE 4:**
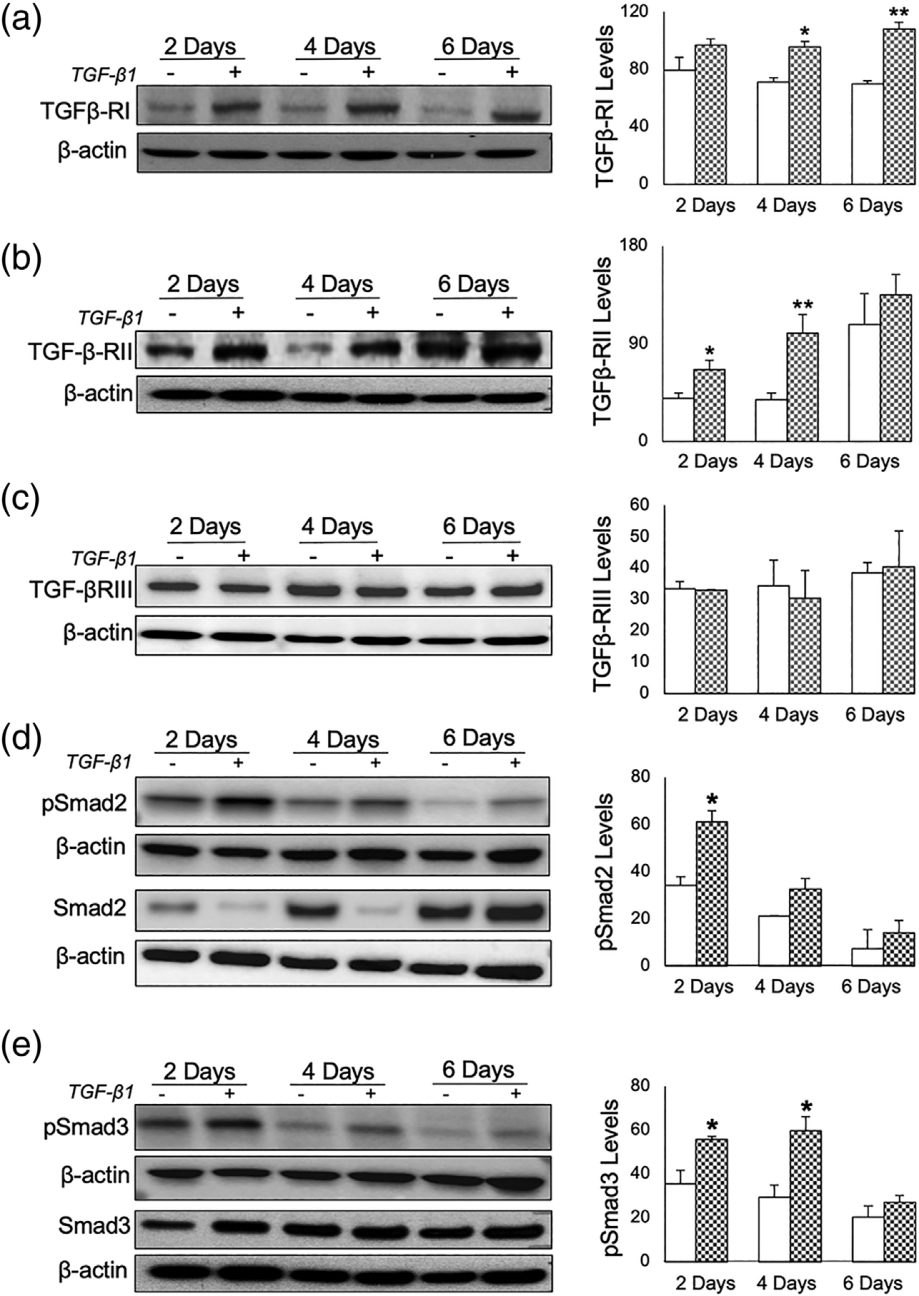
Modulation of the TGF‐β1‐Smad2/3 axis in IMOK cells following chronic TGF‐β1 treatment. Representative western blots and analysis of (a) TGF‐βRI, (b) TGF‐βRII and (c) TGF‐βRIII protein levels in whole lysates prepared from IMOK cells, in the presence or absence of TGF‐β1. (d) Representative western blots and corresponding analyses of phosphorylated and total levels of Smad2 and (e) Smad3 in IMOK cells, in the presence or absence of TGF‐β1. Protein levels were normalized to β‐actin and quantitated by NIH Scion image. Data are presented as mean, SEM. *p < 0.05; **p < 0.01; TGF‐β1 versus control (*n =* 3)

We examined whether chronic TGF‐β1 modulates TGF‐β receptor family members (Miyazono et al., 1993) in IMOK cells by western blotting. TGF‐β1‐treated IMOK cells showed a significant induction of TGF‐βRI and TGF‐βRII expression compared to controls but exhibited no effect on TGF‐βRIII at 2‐, 4‐, or 6‐days (Figure 4a–c). It is well established that TGF‐β1 binds to the TGF‐β receptor complexes to activate downstream Smad signaling (Parikh et al., 2008). Therefore, we next sought to determine whether TGF‐β1 signals via canonical Smad signaling by western blotting in these cells. Our results show a statistically significant increase in phospho‐specific Smad2 levels at 2‐days after TGF‐β1 treatment, whereas no alterations were noted at the 4‐ and 6‐day time‐points (Figure 4d). Similarly, phospho‐Smad3 levels were significantly increased at 2‐ and 4‐days following TGF‐β1 treatment (Figure 4e).

### 3.4 TGF‐β1 upregulation of pluripotency markers in IMOK cells is through TGF‐βRI

**FIGURE 5:**
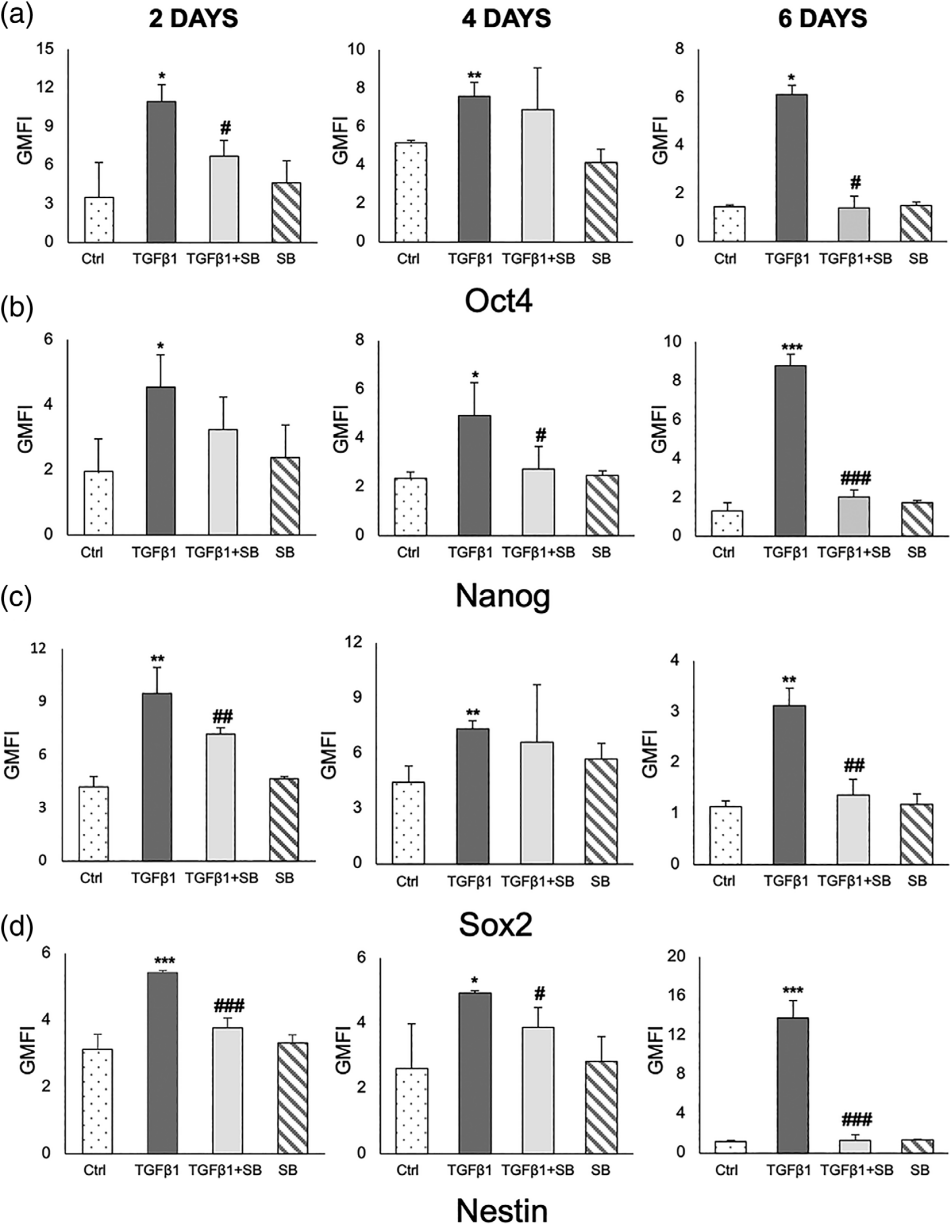
TGF‐β1‐induced stemness is mediated in part by TGFβ1‐RI in IMOK cells in vitro. The stem cell markers (a) Oct4, (b) Nanog (c), Sox2, and (d) Nestin were evaluated by flow cytometry in IMOK cells treated chronically with TGF‐β1, in the presence or absence of the TGF‐β1 receptor inhibitor SB431542 at the indicated time points. Bar charts analyses represent quantification of the GMFI means for each marker in the vehicle‐control (ctrl), TGF‐β1, TGF‐β1 with the TGF‐β receptor inhibitor (TGF‐β1 + SB431542) and the TGF‐β receptor inhibitor (SB431541) only conditions. Results are presented as mean ± SEM. *n =* 3. *Significant compared to control; ^**#**^significant compared to TGF‐β1 treated. Statistical significance was evaluated by student's *t*‐test (*, ^#^ *p* < 0.05; **, ^##^ *p* < 0.01; ***, ^###^ *p* < 0.001)

We explored whether chronic TGF‐β1 induces the upregulation of pluripotency factors in IMOK cells through the TGF‐βRI receptor through the use of the selective antagonist SB431542 as follows: (1) vehicle control; (2) TGF‐β1; (3) TGF‐β1 + SB431542; and (4) SB431542 (10 μM) alone. Flow cytometry analyses confirmed that TGF‐βRI inhibitor SB431542 attenuated the upregulation of pluripotency markers by TGF‐β1 in IMOK cells (Figure 5a–d). Taken together, our data suggest that chronic TGF‐β1, via its cognate receptor TGF‐βRI, can induce stem cell factors in IMOK cells.

## 4 DISCUSSION

A new source of cells with stem‐like properties is required for regenerative dentistry in the future. In the present study, we evaluated whether adult somatic oral epithelial keratinocytes, lining the oral cavity, can be successfully reprogrammed to acquire pluripotent stem cell‐like properties, thereby providing a practically unlimited supply of stem‐like cells that could be differentiated into tissues for regenerative efforts. For example, reprogrammed oral epithelial keratinocytes can serve as the starting cell type in the generation of enamel‐like biomimetic material as recently described (Bazina et al., 2021).

A recent body of evidence suggests that similar to embryonic stem cells, fully differentiated somatic cells can be reprogrammed into a more primitive state, highlighting many questions about the reversible nature of somatic cell reprogramming and induction of pluripotency (Takahashi & Yamanaka, 2006; Yu et al., 2007). Reprogramming adult somatic cells into a pluripotent state can yield iPSCs (Buganim et al., 2013). Intriguingly, iPSCs, which have the capacity to differentiate into multiple cell types, have been generated from adult somatic cells by ectopic expression of an interconnected network of pluripotent transcription factors, including but not limited to, Oct4, Sox2, Klf4, c‐Myc, Nanog or Lin28 (Takahashi et al., 2007; Takahashi & Yamanaka, 2006; Yu et al., 2007). High levels of Oct4 during the initial reprogramming stage was reported as sufficient to generate iPSCs from adult somatic cells (Radzisheuskaya & Silva, 2014). In addition, Oct4 overexpression together with small molecule agents was sufficient to reprogram fibroblasts into iPSCs (Salci et al., 2015) and cells co‐expressing Oct4 and Nanog exhibited a more stable pluripotent state than cells individually expressing these factors alone (Theunissen et al., 2011). Overexpression of Sox2 in mouse and human fully differentiated fibroblasts, was further shown to establish multipotent‐induced neuronal stem cells (Ring et al., 2012). The unique ability of Sox2 to cooperate with Oct4 at specific binding sites is critical for the reprogramming of fully differentiated somatic cells into iPSCs (Aksoy et al., 2013). Thus, together, Oct4 and Sox2 preserve an equilibrium in cell fate decisions, which renders pluripotency (Thomson et al., 2011). Although Nestin is not considered one of the core stem cell transcription factors, human and mouse Nestin‐expressing stem cells in the hair follicle bulge were able to produce neurons, glia, keratinocytes, smooth muscle cells, blood cells and melanocytes in vitro (Yashiro et al., 2015) suggesting a multipotent phenotype with putative therapeutic potential.

Our data demonstrate that chronic TGF‐β1 treatment of the immortalized murine oral epithelial keratinocyte cell line IMOK induced the expression of Oct4, Nanog, Sox2 and Nestin *in vitro*. Their levels of expression varied at different time points, emphasizing the requirement of their sequential induction during the reprogramming of somatic cells to iPSCs (Brambrink et al., 2008). Published evidence describes a later requirement of Nanog and Sox2, than the early requirement for Oct4, to maintain pluripotency (Loh et al., 2006). Our findings are in agreement with previous reports demonstrating an early induction of Oct4, along with later expression of Sox2 and Nanog expression (Jo et al., 2014; Mu et al., 2015). We evidenced Oct4 in IMOK nuclei after TGF‐β1 stimulation, consistent with the known properties of Oct4 in cellular reprogramming and stemness (Oka et al., 2013). Similarly, Nanog was shown to exhibit cellular shuttling, with nuclear localization detected in human embryonic stem cells with transformed Hela and SH‐SY5Y cells displaying cytoplasmic localization (Rodrigo et al., 2017), as well as during enamel organ development (da Cunha et al., 2013).

Furthermore, Hox, Gata and Pax genes, which control sets of genes that define cellular fate conversion during morphogenesis and stem cell self‐renewal were increased by TGF‐β1 (Alharbi et al., 2013; Amsellem et al., 2003; Scialdone et al., 2016). To the best of our knowledge, we are the first to report herein TGF‐β1‐mediated induction of Hox, Pax, and Gata genes has yet to be reported in adult somatic cells elsewhere.

We also report that TGF‐β1 induced the expression of Oct4, Nanog, Sox2 and Nestin in IMOK cells through activation of the TGFβ receptor type I (TGFβR‐I) and downstream Smad2/3 intracellular signaling. Previous reports have shown that SB431542 led to a reduction in Oct4 and Nanog expression, although Nanog expression was more sensitive to this loss of signaling (Greber et al., 2008). In addition, inhibition of TGF‐β1 signaling, via SB‐431542, was able to drastically deprive “stemness features” of glioma‐initiating cells and promote their differentiation (Peñuelas et al., 2009; Valcourt et al., 2005). Additionally, the role of TGF‐β1 in upregulating or maintaining pluripotency markers, including Oct4 and Nanog, through Smad transduction signaling has been reported in a number of transformed cell lines (Bae et al., 2016). Noteworthy, both Oct4 and Nanog also form a complex with Smad2, and/or Smad3 in mouse and human embryonic stem cells (Mullen & Wrana, 2017).

The field of regenerative Dentistry has developed recently and efforts to produce an abundant source of cells for the production of stem‐like cells has been the focus of several investigators. For example, Yamasaki et al. (2014) were successful in developing human pluripotent stem cells using as starting material dental pulp tissue. However, the relative small amount of pulp tissue and the need to sacrifice a healthy tooth for their harvest significantly limits the clinical application of this method. In contrast, the facileness of harvesting adult somatic cells, together with the versatility of generating pluripotent cells with chronic TGF‐β1, opens up exciting opportunities to tackle regenerative challenges linked to the repair of enamel, or other types of mineralized tissues. To this end, IMOK cells reprogrammed by TGF‐β1 have been successfully employed recently in the production of an enamel biomimetic material (Bazina et al., 2021).

## CONFLICT OF INTEREST

U.S. Patent 10,350,147 covering the intellectual property presented herein was issued on July 16, 2019 assigned to The State University of New York Research Foundation, whereby SK and SMB are listed as inventors and therefore have financial interests in royalties and fees.

## AUTHOR CONTRIBUTIONS

Fayrouz Bazina performed most of the experiments detailed herein under the direct supervision of her mentor Sabine M. Brouxhon. Stephanos Kyrkanides served as the principal investigator and inventor, is responsible for the overall project and authored the manuscript.

## Data Availability

The data will be made available upon request.
